# Does wintering north or south of the Sahara correlate with timing and breeding performance in black‐tailed godwits?

**DOI:** 10.1002/ece3.2879

**Published:** 2017-03-21

**Authors:** Rosemarie Kentie, Rocío Marquez‐Ferrando, Jordi Figuerola, Laura Gangoso, Jos C.E.W. Hooijmeijer, A. H. Jelle Loonstra, Frédéric Robin, Mathieu Sarasa, Nathan Senner, Haije Valkema, Mo A. Verhoeven, Theunis Piersma

**Affiliations:** ^1^ Conservation Ecology GroupGroningen Institute for Evolutionary Life Sciences (GELIFES)University of GroningenGroningenThe Netherlands; ^2^ Department of Wetland EcologyDoñana Biological Station (EBD‐CSIC)SevilleSpain; ^3^ CIBER Epidemiología y Salud Pública (CIBER ESP)Spain; ^4^ Ligue pour la Protection des Oiseaux (LPO)Fonderies royalesRochefortFrance; ^5^ Fédération Nationale des ChasseursIssy les Moulineaux CedexFrance; ^6^ Global Flyway NetworkDen BurgTexelThe Netherlands; ^7^ Department of Coastal SystemsNIOZ Royal Netherlands Institute for Sea Research and Utrecht UniversityDen BurgTexelThe Netherlands; ^8^ Present address: Department of ZoologyUniversity of OxfordOxfordOX1 3PSUK

**Keywords:** carryover effect, *limosa limosa*, migration, phenology, repeatability, wintering strategies

## Abstract

Migrating long distances requires time and energy, and may interact with an individual's performance during breeding. These seasonal interactions in migratory animals are best described in populations with disjunct nonbreeding distributions. The black‐tailed godwit (*Limosa limosa limosa*), which breeds in agricultural grasslands in Western Europe, has such a disjunct nonbreeding distribution: The majority spend the nonbreeding season in West Africa, while a growing number winters north of the Sahara on the Iberian Peninsula. To test whether crossing the Sahara has an effect on breeding season phenology and reproductive parameters, we examined differences in the timing of arrival, breeding habitat quality, lay date, egg volume, and daily nest survival among godwits (154 females and 157 males), individually marked in a breeding area in the Netherlands for which wintering destination was known on the basis of resightings. We also examined whether individual repeatability in arrival date differed between birds wintering north or south of the Sahara. Contrary to expectation, godwits wintering south of the Sahara arrived two days earlier and initiated their clutch six days earlier than godwits wintering north of the Sahara. Arrival date was equally repeatable for both groups, and egg volume larger in birds wintering north of the Sahara. Despite these differences, we found no association between wintering location and the quality of breeding habitat or nest survival. This suggests that the crossing of an important ecological barrier and doubling of the migration distance, twice a year, do not have clear negative reproductive consequences for some long‐distance migrants.

## 1 Introduction

Seasonal migration allows animals to exploit areas that are inhospitable at other times of the year (Alerstam, Hedenström, & Åkesson, 2003). Although seasonal migration is widespread throughout the animal kingdom, it is especially well developed in birds (Newton, 2010). In shorebirds, seasonal migration is the rule rather than the exception (Piersma, 2003, 2007), but the distances travelled vary greatly between species. Some species migrate as far as 12,000 km one way (e.g., bar‐tailed godwit *Limosa laponica baueri*: Battley et al., 2012) and cross ecological barriers such as oceans, mountain ranges, and deserts, while others remain close to the breeding grounds, even if that means remaining at sub‐Arctic latitudes (e.g., rock sandpiper *Calidris ptilocnemis ptilocnemis*: Ruthrauff, Dekinga, Gill, & Piersma, 2013). Not only is there considerable among‐species variation in migratory distances, there is also variation among populations within shorebird species (Reneerkens et al., 2009), and even among individuals within the same population (Hooijmeijer et al., 2013; Hötker, 2002).

Migrating long distances requires time and energy that may entail trade‐offs with reproductive events, that is, costs are carried over from one season into the other (Alves et al., 2012; Harrison, Blount, Inger, Norris, & Bearhop, 2011; Klaassen, Lindström, Meltofte, & Piersma, 2001; Norris & Marra, 2007; Senner, Conklin, & Piersma, 2015). This effect may be enhanced when individuals need to cross ecological barriers, because they lack the opportunity to replenish energy during migration (Newton, 2010). Besides avoiding energy costs associated with large displacements, remaining close to the breeding grounds during the nonbreeding season may be advantageous as proximity should enable individuals to better time their arrival and breeding in accordance with local weather conditions (Both, Bouwhuis, Lessells, & Visser, 2006). For instance, in warm springs pied avocets (*Recurvirostra avosetta*) which wintered further away from the breeding grounds arrived later than individuals which wintered nearby (Hötker, 2002). Mistimed arrivals, which is nowadays mainly referring to arriving too late due to global warming (Knudsen et al., 2011), in turn, may compromise the timing of clutch initiation and, consequently, reproductive success (Both et al., 2006).

Because breeding performance can be compared between individuals breeding in the same area but with different migration distances (Ketterson & Nolan, 1983), populations with disjunct nonbreeding ranges allow descriptive tests of possible trade‐offs between migration distance and reproduction. In Eurasian spoonbills (*Platalea leucorodia leucorodia*) breeding in the Netherlands, individuals wintering south of the Sahara have a lower survival probability, breed later, and recruit fewer offspring than birds which remain in Europe (Lok, Overdijk, Tinbergen, & Piersma, 2011; Lok, 2013), suggesting that wintering further away from the breeding grounds and crossing an ecological barrier may be associated with increased fitness costs. Conversely, Icelandic black‐tailed godwits (*Limosa limosa islandica*) wintering in Portugal not only arrive at their breeding sites in Iceland earlier than individuals wintering almost 2,000 km further north in Ireland and England, but also have a higher reproductive success and survival (Alves et al., 2012, 2013). This emphasizes that the fitness costs of migration may be context dependent, for example, whether migration involves crossing an important ecological barrier such as the Sahara Desert (Lok, Overdijk, & Piersma, 2015), alternatively, the quality of the winter site may influence breeding performance.

**Figure 1:**
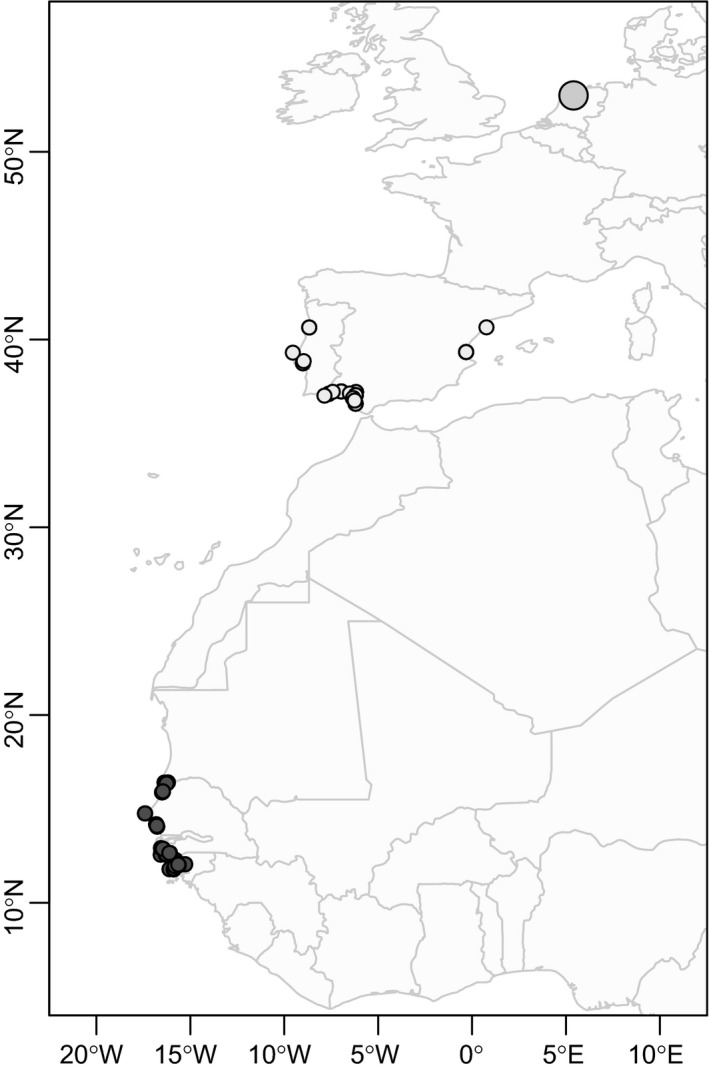
Locations of resightings of color‐marked black‐tailed godwits south of the Sahara in West Africa, September–March (dark gray), and north of the Sahara on the Iberian Peninsula in October (light gray). The Dutch breeding study site is marked with a large gray circle

The great majority of Continental black‐tailed godwits (*Limosa limosa limosa*; hereafter, “godwits”) breed in the Netherlands (Kentie et al., 2016) and spend the nonbreeding season either south of the Sahara—predominantly in the West African countries of Senegal and Guinea‐Bissau (Hooijmeijer et al., 2013)—or north of the Sahara in the Iberian Peninsula (Márquez‐Ferrando, Hooijmeijer, Groen, Piersma, & Figuerola, 2011; see also Figure 1). Although the godwit population has declined by over 75% in the past half‐century (Gill et al., 2007; Kentie et al., 2016), the number of godwits wintering in southern Spain has grown especially in Doñana Wetlands, Spain; the percentage of the flyway population wintering here increased from 4% in the late 1980s to 23% in 2011 (Márquez‐Ferrando, Figuerola, Hooijmeijer, & Piersma, 2014). This increase has been suggested to reflect an increase in the availability of suitable foraging habitat in the form of man‐made fish ponds and irrigated rice fields in the region, at the same time that degradation of wetlands in West Africa has increased (Zwarts, Bijlsma, van Kamp, & Wymenga, 2009). As southern Spain is ~3,000 km closer to Dutch breeding areas and does not necessitate a potentially arduous migration across the Sahara Desert (Klaassen et al., 2014; Lok et al., 2011), it is possible that godwits remaining north of the Sahara may experience higher fitness due to an earlier arrival on the breeding grounds and lower energetic expenditure during migration.

Here we examine whether wintering in distinct regions is related to variation in reproductive parameters in a Dutch breeding godwit population. We examine whether wintering north of the Sahara is advantageous in terms of differences in arrival and laying date, habitat choice, and reproductive parameters. Moreover, we analyzed whether godwits wintering north or south of the Sahara differ in the repeatability of their phenology, exploring whether godwits wintering closer to their breeding area may be more flexible in their timing of arrival. Observational studies linking events during the breeding season with outspoken differences in migration strategies are still thin on the ground (but see Alves et al., 2013; Lok et al., 2011, 2015) and will help determine whether the costs and a different length of migration influences population dynamics in a declining long‐distance migratory bird.

## 2 Materials and methods

### 2.1 Study sites

Since 2004, we monitored godwits breeding in a region of dairy farming in southwest Friesland, the Netherlands (52°55′N, 5°5′E; Kentie, Both, Hooijmeijer, & Piersma, 2015), an area that traditionally held high densities of breeding godwits (Mulder, 1972). From 2004 to 2007, our study area comprised the north of the Workumerwaard, a 300‐ha meadow area largely managed for birds. From 2007 to 2011, our study area was expanded to 8,480 ha of agricultural land surrounding the Workumerwaard, and was expanded again in 2012, with the addition of another adjacent 1,500 ha. Using measures of the occurrence of foot drains, high field water levels, and herb richness (Groen et al., 2012), we categorized all 2,811 fields in the study area as one of two categories reflecting the intensity of agricultural use. “Herb‐rich meadows” represent traditionally managed meadows, often managed especially for birds by postponing mowing until after the incubation period, with the help of agricultural subsidies or as meadow bird reserves, whereas “agricultural monocultures” are industrialized grasslands and arable fields harvested several times a year. We have shown previously that godwits prefer herb‐rich meadows over monocultures (Kentie, Both, Hooijmeijer, & Piersma, 2014) and that godwits breeding in herb‐rich meadows have higher nesting success and chick survival than godwits breeding in monocultures (Kentie, Hooijmeijer, Trimbos, Groen, & Piersma, 2013; Kentie et al., 2015). On this basis, we consider herb‐rich meadows to be the higher‐quality breeding habitat.

Godwits spend their winter either north or south of the Sahara Desert (Figure 1). During northward migration, the majority of godwits stage at one (or more) of three sites on the Iberian Peninsula: (1) Doñana Wetlands, Spain; (2) the rice fields of central Extremadura, Spain; and (3) the Tejo and Sado Estuaries, Portugal (Alves, Lourenço, Piersma, Sutherland, & Gill, 2010; Lourenço, Mandema, Hooijmeijer, Granadeiro, & Piersma, 2010; Márquez‐Ferrando et al., 2014; Masero et al., 2010). We have systematically monitored godwit flocks in Extremadura and the Tejo and Sado estuaries for color‐marked godwits in late winter since 2006 (Lourenço, Kentie, et al., 2010; Masero et al., 2010). In November 2010, we also began surveying Doñana and the surrounding region for color‐marked godwits; since 2011, the resighting intensity has increased to a minimum of biweekly surveys each winter from October–March (Márquez‐Ferrando et al., 2011). All sightings in Doñana from before 2010 were from other experienced observers and not part of systematic surveys. Our coverage of West African wintering sites has been less consistent and intense: In 2009, the rice fields of Guinea‐Bissau and Senegal were searched; subsequent expeditions were held in November 2014, and August and December 2015. In addition to the resightings made by members of dedicated expeditions, we also included resightings from volunteers across the flyway.

### 2.2 Defining an individual's wintering region

Data from 82 satellite transmitters and three geolocation tracking devices mounted on adult godwits both in a Spanish spring staging area (see Senner, Verhoeven, Abad‐Gómez, et al., 2015) and on the breeding grounds (unpubl. data T. Piersma, stored at www.movebank.org and viewable at volg.keningfanegreide.nl) revealed that adult godwits were faithful to wintering location at the regional level. We thus classified an individual as wintering south of the Sahara if it was seen in West Africa at least once in the nonbreeding period (July–March) during any of the years of our study (2004–2015). Because godwits migrating to and from West Africa also use Doñana and other regions of the Iberian Peninsula as a stopover location during late summer (June–September; Hooijmeijer et al., 2013) and winter to early spring (November–March; Márquez‐Ferrando et al., 2014), we classified an individual as wintering north of the Sahara if it was resighted in October during any year (2007–2015). Finally, young birds in general may be less faithful to both wintering and breeding sites (e.g., Lok et al., 2011) and may differ in phenology and breeding output from adult birds. We therefore only used sightings of godwits caught in the Netherlands as adults, or, for birds marked as chicks, only after they were older than 3 years (see Table 1 for sample sizes).

**Table 1:** Sample sizes of total observations (Total *N*) and of uniquely color‐marked individual black‐tailed godwits (Ind) wintering north and south of the Sahara used in the analyses. The sample of arrival dates is reported for females and males; nest success is reported for nests located in low‐ and high‐quality habitats; observations of breeding habitat choice excluding duplicate observations of individuals that did not change their habitat

		Arrival date		Lay date		Egg volume		Breeding habitat		Nest success	
Region	Sex	Total *N*	Ind	Total *N*	Ind	Total *N*	Ind	High	Low	Total *N*	Ind
North	F	264	93	173	89	171	88	112	59	81	60
	M	293	87	–	–	–	–	110	34	192	111
South	F	160	61	113	61	61	113	82	30	59	40
	M	190	70	–	–	–	–	82	33	147	89

### 2.3 Measuring breeding parameters

While our study began in 2004, we only used data on breeding parameters from 2007 onwards, as the study area prior to 2007 was largely composed of herb‐rich meadows; we did, however, include birds marked prior to 2007. Each year (2007–2015), we systematically searched the breeding study area and surroundings daily for color‐marked godwits, from the arrival of the first adults in early March until the beginning of the laying phase in April. Resighting probability was high, with on average 3 ± 1 days between resightings (Senner, Verhoeven, Abad‐Gómez, et al., 2015). We used the date of the first observation of an individual at the breeding area in a given year as its arrival date. We confined our analyses to individuals first seen before 30 April, as our resighting effort decreased after this point in order to focus on monitoring nests. During the laying and incubation phases between April and June, we surveyed the study area for finding nests by walking side‐by‐side through meadows to locate nests during the laying phase or, later, by flushing incubating adults from nests. When a nest was found, we measured each egg's width and length to the nearest mm. Godwits, like other shorebirds, usually have a clutch size of four eggs (Arnold, 1999). We used the egg flotation method (van Paassen, Veldman, & Beintema, 1984) to age the nest and predict its laying and hatching date (25 days after laying the first egg). Egg volume was estimated with the formula: length × width^2^ × 0.524 (from Romanoff & Romanoff, 1949). We identified the parents of the nest by reading color rings with a spotting scope, or with camera traps. We considered a nest successful if at least one chick was found in the nest, or, as chicks leave the nest within 24 hr (Lind, 1961), if we found an indication of successful hatching such as broken eggs without blood or yolk and membranes clearly visible, and tiny egg fragments in the bottom of the nest. A nest was considered unsuccessful if we found all eggs abandoned, egg remains without membranes, egg remains with yolk or blood, or an empty nest without any egg remains.

To prevent nest abandonment, and because the birds are easier to capture close to hatch, parents were caught at the end of the incubation stage. Adults were either caught in a walk‐in‐trap, with a mist net placed over the nest, or occasionally by hand from the nest. Each individual was uniquely marked with four plastic color rings, a colored flag, and numbered metal ring. We measured bills and tarsus length to the nearest 0.1 mm, and head and wing length, and the length of the tarsus plus mid‐toe without nail to the nearest mm. Prefledging chicks of >10 days were considered large enough to wear a color ring combination, although few were captured because of the difficulty locating them. We also collected a blood sample (30 μl from the brachial or tarsal vein) from each individual for molecular sexing (see Trimbos et al., 2011); when a blood sample was lacking, we used morphological measurements (see Schroeder et al., 2008) to sex the remaining 11% of the birds. This work was done under license numbers 4339E and 6350A following the Dutch Animal Welfare Act Articles 9 and 11.

### 2.4 Data analyses

To examine whether arrival date at the breeding area differed between individuals wintering north or south of the Sahara, we used a linear mixed‐effect model (LMM) with a normal error structure. We included sex and its interaction with wintering region as factors, because males normally arrive earlier than females (Lourenço et al., 2011). Individual and year were included as random terms. Although resighting probability of arriving godwits is lower than one, we do not expect differences in resighting probability between the two groups.

We then examined whether wintering north or south of the Sahara correlated with the probability to breed in high‐quality breeding habitat. Although we have observations for 13% of the individuals from multiple years, a generalized linear mixed‐effect model (GLMM) with year and individual as a random effect did not converge because too few individuals changed breeding habitats between years. To overcome this problem, we used a generalized linear model (GLM) with the proportion of breeding attempts in good quality areas per individual over the years (with the function cbind()). We used a binomial error structure and a logit link function.

Next, we determined whether lay dates of females differed between individuals from the two wintering regions with a LMM with a normal error structure and individual and year as random terms. Godwits can lay a second clutch when the first failed (Senner, Verhoeven, Hooijmeijer, & Piersma, 2015), and we used the lay date of first nest if we knew of other attempts. However, for the majority of nests we were unable to correct for repeat nesting attempts because, in most cases, a pair's full nesting history was unknown. If nest success were to differ between groups, the group with the lowest nest success would likely have more second nesting attempts, potentially biasing our estimates of lay dates if we missed a large number of first attempts. However, there was no effect of wintering region on nest success (see Section 3).

We then tested whether the average egg volume of a clutch differed between females from different wintering regions with a LMM with a normal error structure and individual and year as random terms. We included lay date in the model because a previous study found an effect of lay date (in some years) on egg volume (Lourenço et al., 2011), and included an interaction between lay date and wintering region. Because female size may influence egg size, we checked with an ANOVA whether females wintering north or south of the Sahara differed in structural body size. Structural female size was described by the first component of a principal component analysis in which we added the biometric variables (bill length, total head length, wing length, tarsus length, tarsus‐to‐toe length). This principal component explained 51% of the variation.

We calculated the individual repeatability for arrival date by fitting a LMM with individual as random term. Repeatability is the between‐individual variation divided by the sum of between‐individual variance and residual variance (see Nakagawa & Schielzeth, 2010). We did this separately for each wintering region. We then compared the 95% confidence intervals of the repeatability of both wintering regions.

We also tested for an effect of wintering region on daily nest survival probability in a mark–recapture framework, because it accounts for nests not found before they were lost (Dinsmore, White, & Knopf, 2002). In the model in which we estimated the effect of wintering region on nest success, we always included year due to known strong year effects (Kentie et al., 2015), and tested for an effect of sex, and the interaction between sex and wintering region. We knew the wintering location of both adults of 29 of 532 nests with identified adults (belonging to 16 pairs; godwits are monogamous, see Kentie et al. (2014)). If the wintering locations of both adults from a nest were known, we randomly chose one of the adults for inclusion in our analyses. Of these 16 pairs, in six cases one partner wintered south while the other wintered north of the Sahara.

We used Program R (v. 3.1.1; R Core Team 2014) with the packages “lme4” (Bates, Maechler, Bolker, & Walker, 2014) for LMM, and “RMark” (Laake, 2013) for daily nest survival analysis, and “rptR” using 10,000 iterations to estimate repeatability (Schielzeth, Stoffel, & Nakagawa, 2016). Arrival date and lay date were log‐transformed after visually checking residual plots. For the LMMs, we calculated the significance of the fixed and random effects, and the 95% confidence intervals for parameters with a parametric bootstrap (1,000 iterations). Fixed covariates were excluded from the fullest model in a stepwise backward elimination if they failed to explain variation (*p* > .05). Models of daily nest survival were compared with the second‐order AIC (Akaike information criterion) for small samples (AICc) (Burnham & Anderson, 2002).

## 3 Results

**Figure 2:**
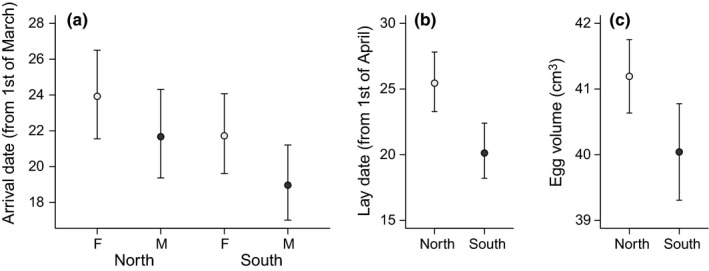
Arrival dates (a), lay dates (b), and egg volume (c) of black‐tailed godwits wintering north or south of the Sahara. The mean and 95% confidence intervals are shown. For individuals with data for several years, we used the mean to avoid pseudoreplication. Lay date and egg volume are reported only for females

Male and female godwits were found in equal proportions to winter north or south of the Sahara (χ^2^ = 0.277, df = 1, *p* = .60, *N* = 495, Table 1). Also, godwits with a known age, either younger than or older than three when first seen on the wintering grounds, had similar chances to be seen north or south of the Sahara (χ^2^ = 0.111, df = 1, *p* = .74, *N* = 149). Males wintering south of the Sahara arrived on average at the Dutch breeding areas on 19 March (17–21 March, 95% CI), while northerly wintering males arrived on average on 21 March (19–24 March, 95% CI). For females, the average arrival dates were 22 March (20–24 March, 95% CI) and 24 March (22–27 March, 95% CI), respectively. These differences in arrival date were significant, and males arrived significantly earlier than females (Figure 2a, Table 2a). The interaction between wintering region and sex was not significant. The repeatability of arrival dates was similar between godwits wintering south of the Sahara (0.24, 0.12–0.36 95% CI) and north of the Sahara (0.24, 0.14–0.33 95% CI).

**Table 2:** Results of mixed‐models testing for the effects of wintering region on arrival date, lay date, egg volume, proportion of godwits breeding in the good quality habitat (herb‐rich meadows) of black‐tailed godwits wintering either north or south of the Sahara

Response variables and random effects	Predictors	Estimate	*SE*	*p*
(a) Arrival date (counted from 1 March)	Intercept	3.186	0.050	<.01
	Wintering region	*−*0.122	0.040	<.01
	Sex	*−*0.112	0.040	<.01
	*Wintering region × sex*	*−0.032*	*0.079*	*.69*
Individual		0.232	0.020	<.01
Year		0.103	0.035	<.01
Residual		0.393	0.011	<.01
(b) Laying date (counted from 1 April)	Intercept	3.236	0.047	<.01
	Wintering region	*−*0.238	0.056	<.01
Individual		0.241	0.028	<.01
Year		0.079	0.036	<.01
Residual		0.286	0.018	<.01
(c) Egg volume	Intercept	41.296	0.329	<.01
	Wintering region	*−*1.194	0.451	<.01
	*Lay date (centered)*	*−0.010*	*0.014*	*.49*
	*Wintering region × lay date*	*0.039*	*0.273*	*.19*
Individual		2.457	0.175	<.01
Year		0.338	0.184	.01
Residual		1.408	0.085	<.01
(d) Proportion breeding good habitat	Intercept	0.870	0.124	<.01
	Wintering region	0.087	0.193	.65
	*Sex*	*0.270*	*0.191*	*.16*
	*Wintering region × sex*	*−0.629*	*0.390*	*.11*

Reference level for wintering region is “north of Sahara” and “female” for sex. Variables removed from the final model are in italics. Arrival and lay dates were log‐transformed, and breeding habitat and nest success are on a logit scale. Random effect estimates refer to standard deviations.

Females that wintered south of the Sahara on average initiated clutches on 20 April, which was 5 days (3–8 days, 95% CI) earlier than females wintering north of the Sahara (Figure 2b, Table 2b). Although early laying females generally tend to lay larger eggs (Schroeder et al., 2012), in our study females wintering south of the Sahara laid smaller eggs: Egg volume differed by 3% between wintering regions, with females wintering south of the Sahara laying eggs of 40.1 cm^3^ (39.4–40.9 95% CI) and females wintering north of the Sahara laying eggs of 41.3 cm^3^ (40.7–41.9 95% CI; Figure 2c, Table 2c). The interaction between lay date and wintering region had no significant effect on egg volume. Females wintering north or south of the Sahara did not differ in structural body size (*F*_1,187_ = 0.038, *p* > .5).

On average, an individual had a 71% (67–75 95% CI) chance to be found in herb‐rich meadows, and wintering region and sex had no effect on this probability (Table 2d). Daily nest survival rates varied among years, ranging between 0.981 in 2015 and 0.998 in 2013. However, daily nest survival did not differ for birds from the two wintering regions (Table 3; ∆AICc = 1.9, but with one additional parameter not competitive (Arnold, 2010)), or among sexes from the two regions either (∆AICc = 0.6, but again not competitive with one additional parameter). This analysis only involved nests for which an adult with known wintering region was identified, biasing the daily nest survival toward higher probabilities as we could not always immediately identify the attending adults when the nest was found, thus potentially excluding predated/abandoned nests before adult identification was possible.

**Table 3:** Model selection results for daily nest survival analysis as function of wintering region (wint), year, sex, and the interaction between wintering region and sex of black‐tailed godwits wintering either north or south of the Sahara. The most parsimonious model is shown in bold

Model	*k*	AICc	∆AICc	Weight	Deviance
**year**	**9**	**659.91**	**0.00**	**0.40**	**641.89**
	10	660.52	0.61	0.29	640.49
wint + year	10	661.85	1.93	0.15	641.82
wint + sex + year	11	662.41	2.50	0.11	640.38
wint*sex + year	12	664.34	4.43	0.04	640.30

*k* is number of parameters.

## 4 Discussion

Like several other European migrant birds (e.g., Flack et al., 2016; Lok et al., 2011; Reneerkens et al., 2009), Continental black‐tailed godwits have a disjunct wintering area and can either be found north or south of the Sahara Desert. Godwits migrating to the southern wintering region cross the Sahara Desert twice and a return trip covers a total (beeline) distance of ~10,000 km, while godwits remaining at the northern location travel less than half the distance: ~4,000 km. We predicted that, because of their longer flight and the lack of stopover sites in the Sahara, godwits wintering furthest south would arrive later because they would need more time replenishing their energy at the staging sites in southern Spain and Portugal. Yet, godwits wintering south of the Sahara arrived two days earlier and initiated their clutch five days earlier than godwits wintering north of the Sahara. The eggs of godwits wintering north of the Sahara, however, were 3% larger than eggs of godwits wintering south of the Sahara, which could not be explained by body size differences; females wintering north and south of the Sahara were of similar structural size. Although southern wintering godwits arrived earlier, we found no relationship between wintering location and habitat quality of the acquired territory, or with daily nest survival probability.

Previous studies of other long‐distance migratory birds with similarly disjunct populations have found a positive correlation between migration distance and the timing of spring arrival at the breeding grounds, and timing of clutch initiation (i.e., Bregnballe, Frederiksen, & Gregersen, 2006; Lok, 2013; Woodworth et al., 2016). We were surprised to find that godwits wintering south of the Sahara arrived and laid their clutches earlier than godwits wintering north of the Sahara. However, a recent study involving the closely related Icelandic black‐tailed godwit found that individual arrival dates were primarily related to breeding and wintering habitat quality, which resulted in individuals wintering furthest away arriving earliest in Iceland (Alves et al., 2012; Gunnarsson et al., 2006). In combination, these studies suggest that the effect of migration on phenology and reproduction is species and context dependent and should not a priori be assumed to be greater for long‐distance migrants.

Does earlier arrival and clutch laying in our study population suggest that wintering in West Africa is more favorable for Continental godwits than wintering in Southern Spain, for instance because of environmental related differences of the two regions? Despite arriving earlier, which would in principle give them first access to high‐quality breeding territories, godwits wintering south of the Sahara did not occupy a higher proportion of the better breeding habitat. Moreover, although previous studies on godwits have found that early lay dates are associated with higher reproductive success (Kentie et al., 2015; Lourenço et al., 2011; Schroeder, Hooijmeijer, Both, & Piersma, 2006), we found no indication that birds wintering in West Africa had higher nest success. On the contrary, females wintering north of the Sahara laid larger eggs than females wintering south of the Sahara. In general, larger eggs hatch chicks of better quality (Krist, 2011) and, in godwits, egg volume is strongly correlated with hatchling mass, which is in turn correlated with chick survival (Hegyi & Sasvari, 1998; Schroeder et al., 2012). This might suggest a trade‐off between early arrival, or a longer migration, and maternal investment in eggs. Unfortunately, our study was unable to examine the potential longer‐term consequences of these differences because the sample size of recruits was too low for sufficient statistical power. Thus, it remains to be tested whether laying earlier and a difference of 3% in egg size represents a significant biological trade‐off, and whether it is enough to affect chick condition or survival.

Repeatability in timing of migratory movements is generally high among long‐distance migrants (Conklin, Battley, & Potter, 2013; Gill et al., 2014; Senner, Hochachka, Fox, & Afanasyev, 2014). We predicted that, as in pied avocets (Hötker, 2002), godwits wintering closer to the breeding grounds would vary more strongly in their arrival and therefore lay dates, as they would be better able to respond to changing local conditions at the breeding grounds. However, we found that arrival dates were similarly repeatable among individuals for individuals wintering north and south of the Sahara, and comparable with a previous study on repeatability in arrival date of this study population (Lourenço et al., 2011; between 0.18 and 0.29). The repeatability of arrival dates is likely to be underestimated, as not all individuals would have been seen immediately upon arrival. This repeatability in arrival dates in both groups does not suggest differences in the ability to predict conditions on the breeding grounds (see Winkler et al., 2014 for a discussion of this cognitive process). This is corroborated by the fact that during a spring with extremely cold temperatures, godwits departed from their Iberian staging areas at the same dates as in other years (Senner, Verhoeven, Abad‐Gómez, et al., 2015). Nonetheless, all individuals were flexible enough to respond in such a way that reproductive success was not affected (Senner, Verhoeven, Abad‐Gómez, et al., 2015).

There is thus no clear evidence to suggest that the recent and rapid growth of the Iberian nonbreeding population (Márquez‐Ferrando et al., 2014) is associated with differential reproductive success related to choice of wintering sites or migration distance. Perhaps the increasing proportion of birds remaining north of the Sahara is better explained by higher survival, as the mortality rate of godwits equipped with satellite tags was highest during the crossing of the Sahara Desert (unplubl. data). In any case, the costs of migration and consequences of choosing among different wintering sites for godwits, and other species, require further study to fully understand and predict the ecological responses of long‐distance migrants to environmental changes (Webster, Marra, Haig, Bensch, & Holmes, 2002). This is especially the case in a system in which some individuals cross a barrier such as the Sahara, which may increase their mortality rate in years with more hazardous wind and weather conditions (Klaassen et al., 2014; Lok et al., 2015), but provide them with potential benefits (e.g., early arrival) in other years. Ideally, future studies will incorporate site‐specific seasonal survival estimates (see Piersma et al., 2016; Rakhimberdiev, van den Hout, Brugge, Spaans, & Piersma, 2015) and consider habitat quality at nonbreeding sites. Such work will help to elucidate how migration influences patterns of demography, biogeography, and the diversity of migratory strategies used by birds.

## Conflict of Interest

None declared.
